# Supplementary material for: Refined characterization of circulating tumor DNA through biological feature integration
Source: Sci Rep. 2022 Feb 4;12:1928. doi: 10.1038/s41598-022-05606-z (PMC8816939; doi:10.1038/s41598-022-05606-z)
Supplement: Supplementary file 1 — Supplementary Information 1. [file 41598_2022_5606_MOESM1_ESM.docx]

Supplementary Materials for

**Refined characterization of circulating tumor DNA through biological feature integration**

Havell Markus, Dineika Chandrananda, Elizabeth Moore, Florent Mouliere, James Morris, James Brenton, Christopher G. Smith, and Nitzan Rosenfeld

To whom correspondence should be addressed: nitzan.rosenfeld@cruk.cam.ac.uk

**Supplementary Figures:**


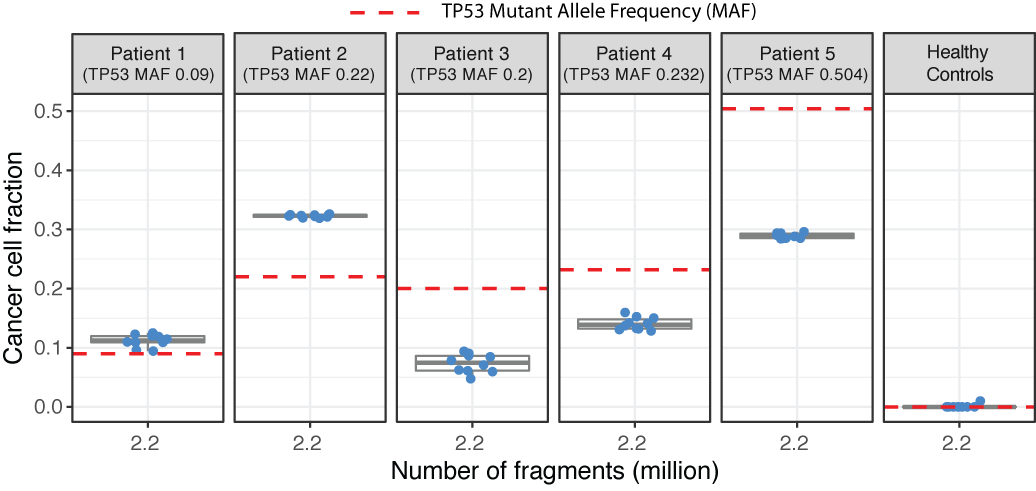


**Figure S1.** The cancer cell fraction (CCF) as estimated by copy number quantification analysis of 10 replicates from each patient and control panel. The red dashed horizontal line marks the TP53 mutant allele fraction measured using targeted amplicon sequencing.


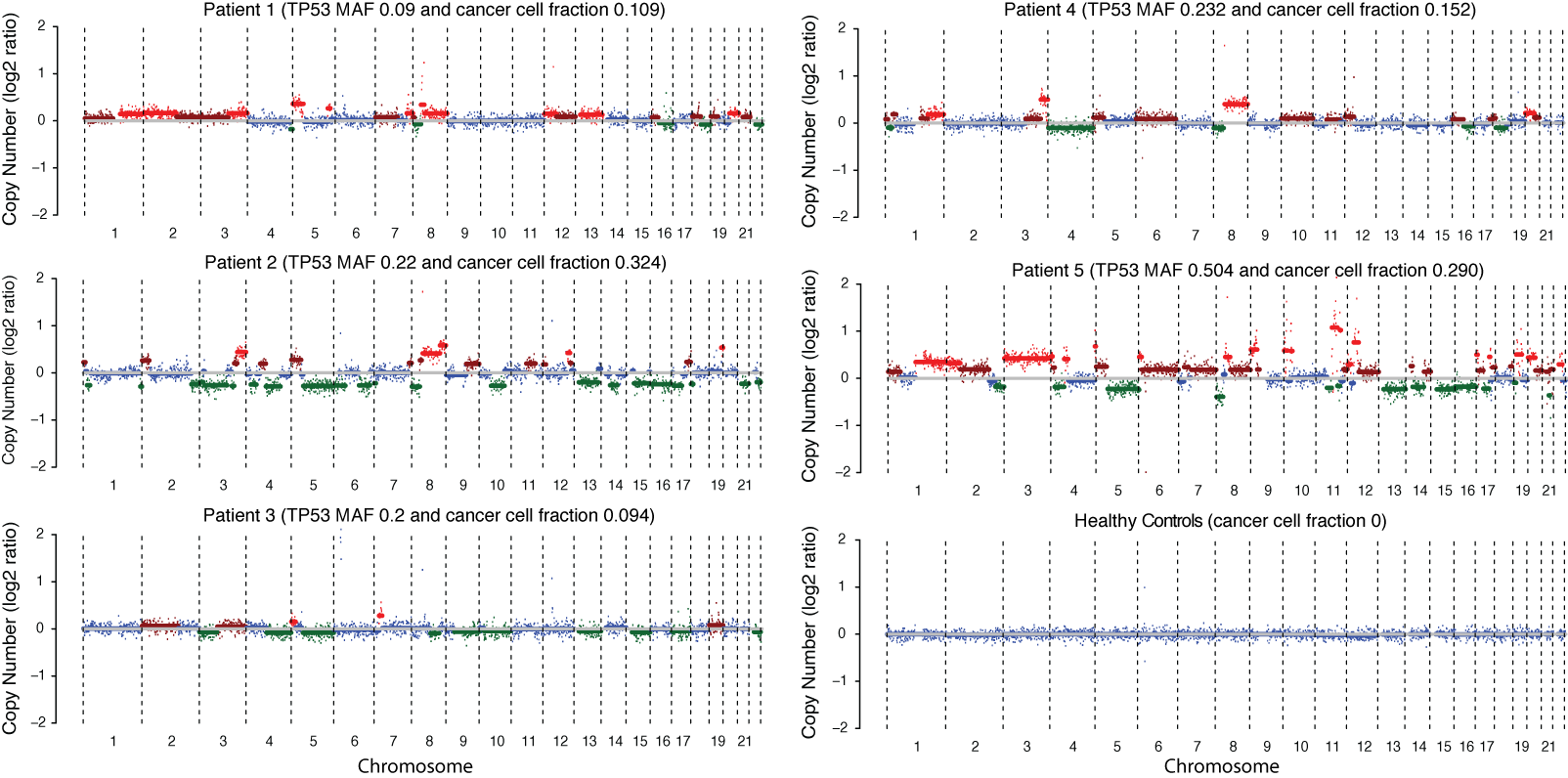


**Figure S2.** Copy number profiles of the 5 HGSOC patients and panel of healthy controls before any feature selection using shallow whole-genome sequencing at 0.1x coverage (2.2 million fragments).

**Figure S3.** The average relative copy number values from the 10 replicates of each patient. The different colors indicate results from using fragments with no size selection, and those with fragment length 240-324bp and 172-239bp.


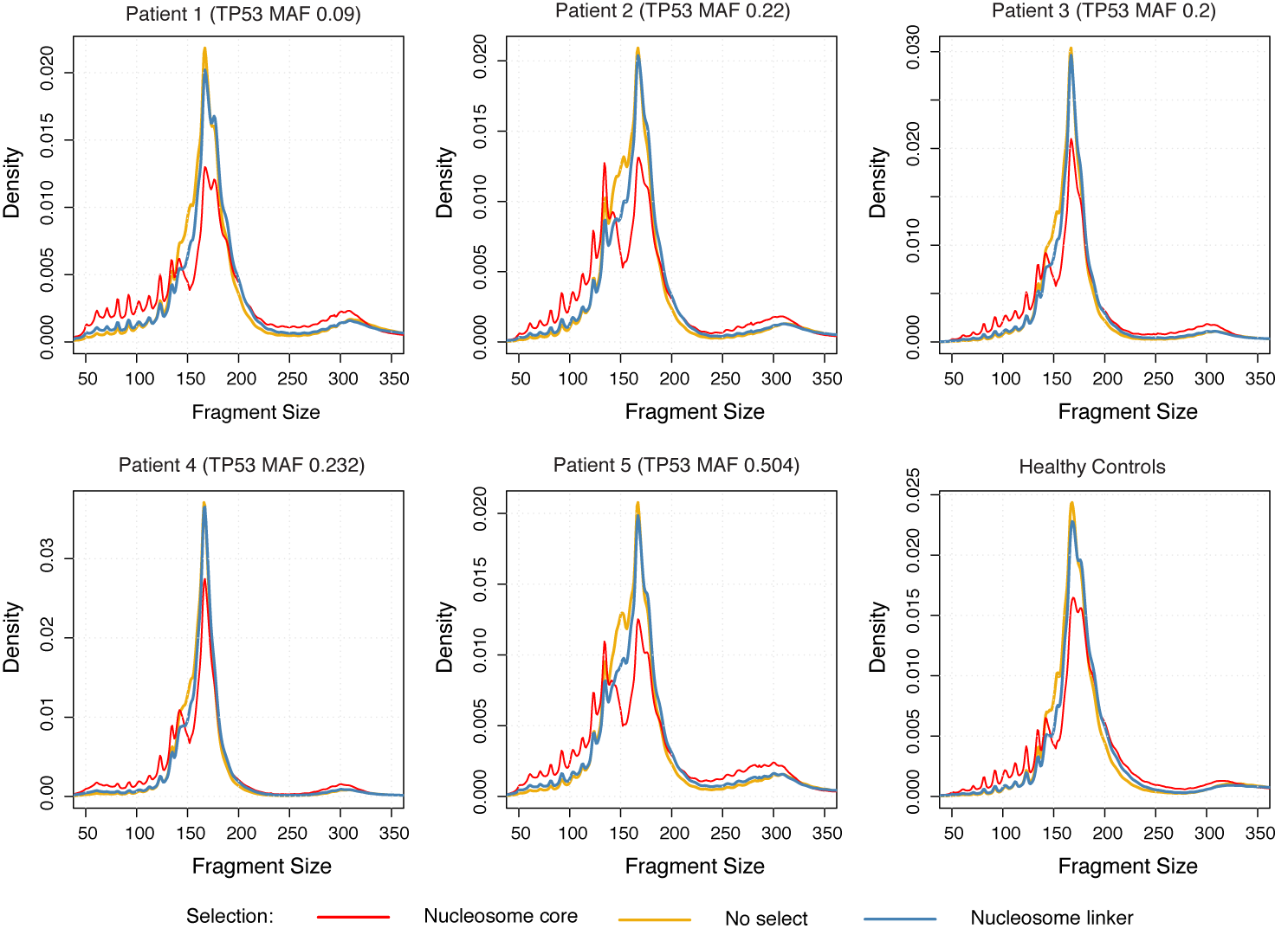


**Figure S4.** Fragment size distribution of fragments with no size selection, fragments that start and end within nucleosome core region, and fragments that start and end within nucleosome linker region.


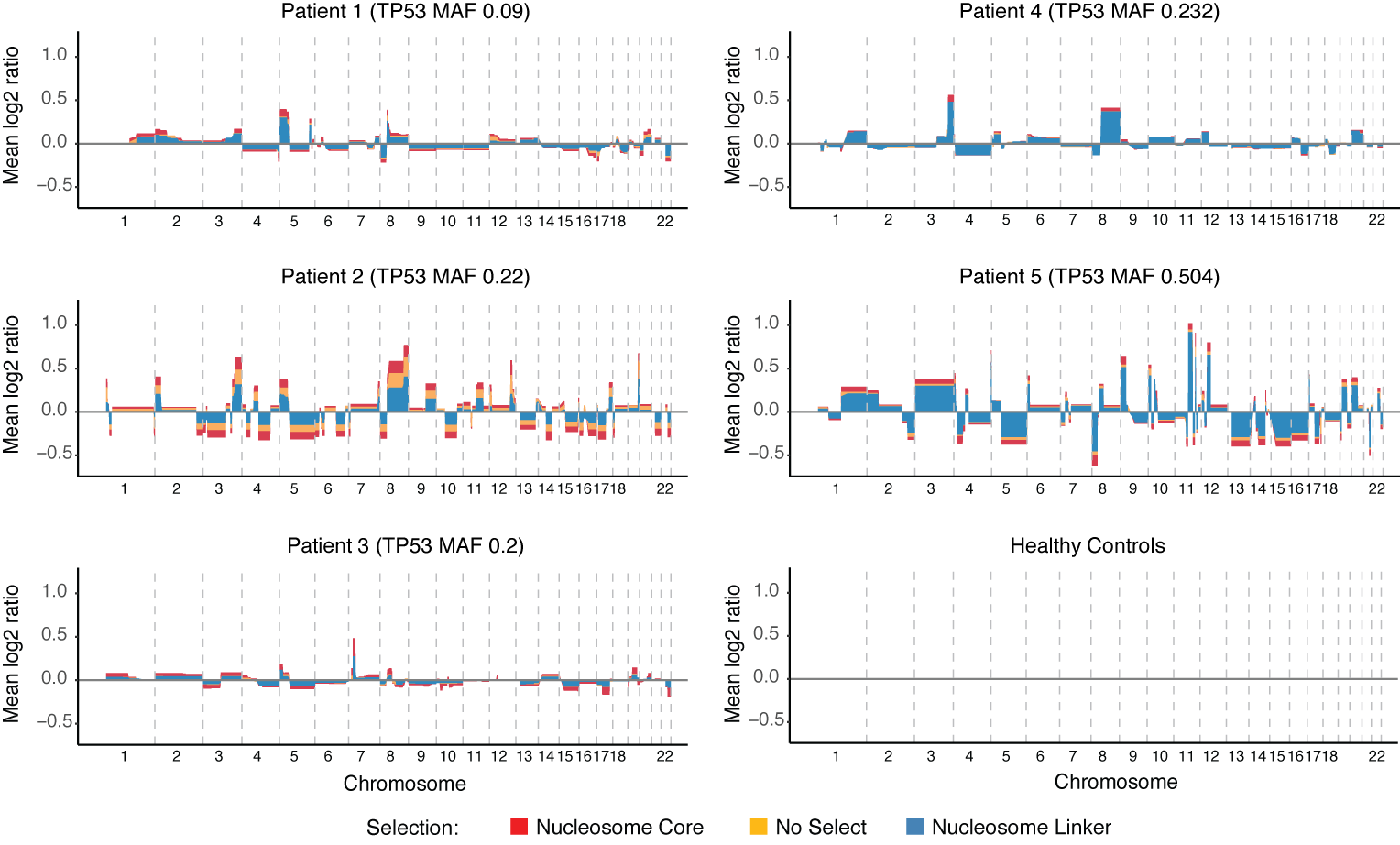


**Figure S5.** The average relative copy number values from the 10 replicates of each patient. The different colors indicate results from using fragments with no size selection, fragments that start and end within nucleosome core region, and fragments that start and end within nucleosome linker region.

**Figure S6.** Per base mean mono-nucleotide frequency of 10bp downstream and upstream of fragment start sites. Calculated on a set of 3 million fragments randomly selected 10 times from the panel of healthy controls and HGSOC Patient 5.

**Figure S7.** Per base mean mono-nucleotide frequency of 10bp downstream and upstream of fragment end sites. Calculated on a set of 3 million fragments randomly selected 10 times from the panel of healthy controls and HGSOC Patient 5.

**Figure S8.** Per base mean di-nucleotide frequency of 10bp downstream and upstream of fragment start sites. Calculated on a set of 3 million fragments randomly selected 10 times from the panel of healthy controls and HGSOC Patient 5.

**Figure S9.** Per base mean di-nucleotide frequency of 10bp downstream and upstream of fragment end sites. Calculated on a set of 3 million fragments randomly selected 10 times from the panel of healthy controls and HGSOC Patient 5.

**Figure S10.** Per base mean tri-nucleotide frequency of 10bp downstream and upstream of fragment start sites. Calculated on a set of 3 million fragments randomly selected 10 times from the panel of healthy controls and HGSOC Patient 5.

**Figure S11.** Per base mean tri-nucleotide frequency of 10bp downstream and upstream of fragment end sites. Calculated on a set of 3 million fragments randomly selected 10 times from the panel of healthy controls and HGSOC Patient 5.


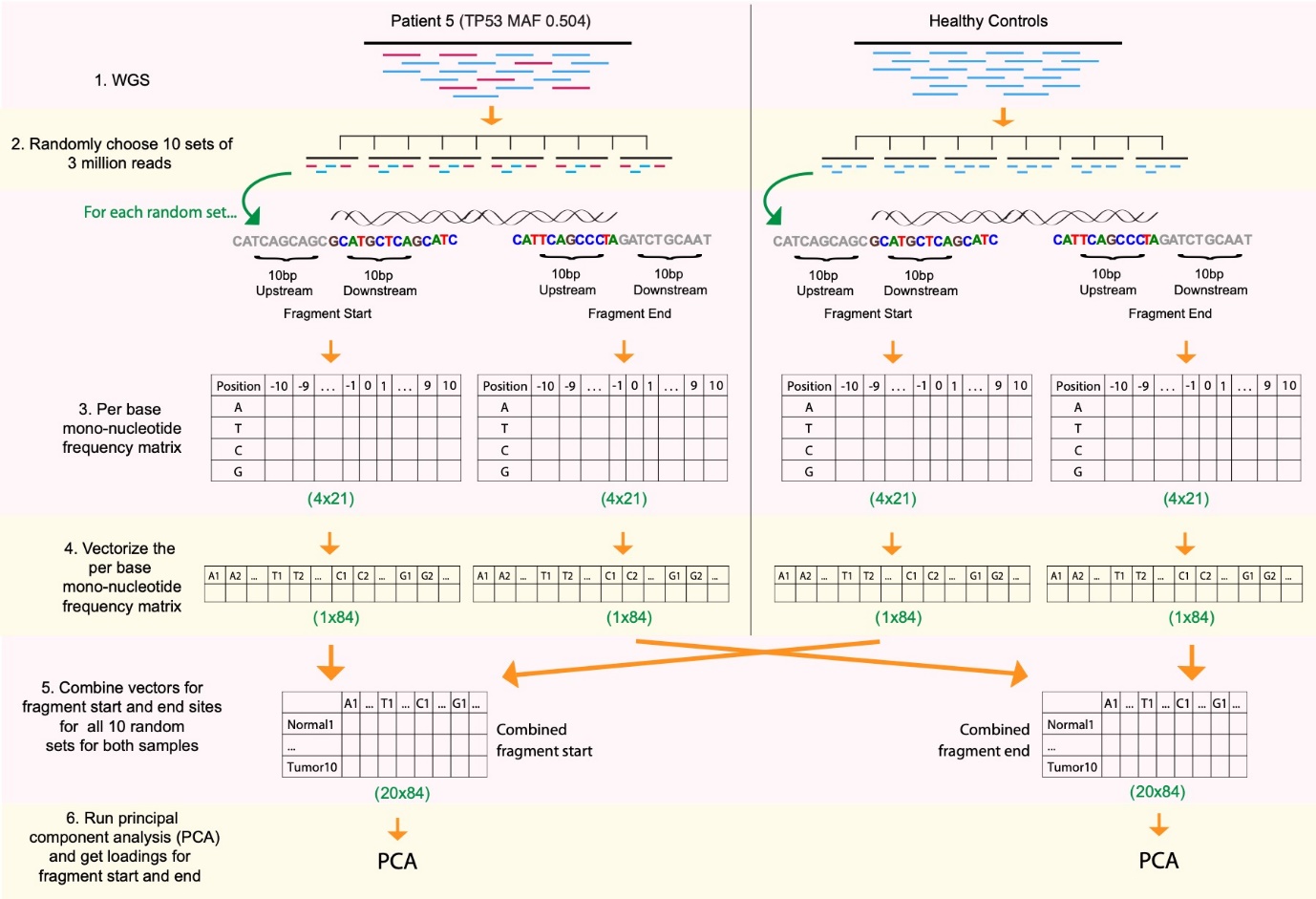


**Figure S12.** Schematic representation of the first phase to calculate the mono-nucleotide frequency score. Similar steps were taken to calculate the di- and tri- nucleotide frequency scores.

**Figure S13.** Steps to calculate nucleotide motif scores. (A) 10 technical replicates were created for the panel of healthy controls and Patient 5 by randomly selecting 3 million fragments. For each replicate, 6 position weight matrices (PWM) were constructed describing the per base mono-, di-, and tri- nucleotide frequencies of the genomic sequences flanking 10bp downstream and upstream of both fragment start and end sites. Principal component analysis was then carried out on the collapsed and concatenated mono-, di-, and tri- nucleotide PWM from all 10 technical replicates of healthy controls and Patient 5 (See Supplementary Figure 4 for more details). All replicates from the panel of healthy controls and Patient 5 clustered separately in all 3 matrices that summarized the mono-, di-, and tri- nucleotide frequencies of fragment start sites. Data for fragment end sites are not shown, however similar clustering of both sample types was observed. The loading vectors of fragment start sites for mono-, di-, and tri- nucleotide frequencies are also shown in each plot. (B) Schematic representation of how to calculate the mono-nucleotide motif score for an individual fragment using the principal component loading vectors of fragment start and end site mono-nucleotide frequencies. For a given fragment, two binary per base mono-nucleotide fragments are created that indicate whether a given nucleotide is present at a given position of fragment start and end site. Then the mono-nucleotide motif score is calculated by the sum of the dot product of binary mono-nucleotide vector of fragment start and end site with the principal component loading vector of fragment start and end site mono-nucleotide frequencies. Similar steps were taken to calculate the di- and tri- nucleotide motif scores. (C) The distribution of per base tri-nucleotide bias score for all fragments of 5 HGSOC patients and panel of healthy controls.

**Figure S14.** The average relative copy number values from the 10 replicates of each patient. The different colors indicate results from fragments with no selection, fragments with per base tri-nucleotide bias score (PBTNB score) less than or equal to -0.3 and greater than or equal to 0.3.

**Figure S15.** Fragment size distribution of fragments with no size selection, fragments with tri-nucleotide motif score less than or equal to -0.3 and greater than or equal to 0.3.
